# Barriers to the widespread adoption of diagnostic artificial intelligence for preventing antimicrobial resistance

**DOI:** 10.1038/s41598-025-95110-x

**Published:** 2025-04-16

**Authors:** Hiromu Ito, Takayuki Wada, Genki Ichinose, Jun Tanimoto, Jin Yoshimura, Taro Yamamoto, Satoru Morita

**Affiliations:** 1 Department of International Health and Medical Anthropology, Institute of Tropical Medicine, Nagasaki University, Nagasaki, Japan; 2 Graduate School of Human Life and Ecology, Osaka Metropolitan University, Osaka, Japan; 3 Osaka International Research Center for Infectious Diseases, Osaka Metropolitan University, Osaka, Japan; 4 Department of Mathematical and Systems Engineering, Shizuoka University, Shizuoka, Japan; 5 Department of Energy and Environmental Engineering, Interdisciplinary Graduate School of Engineering Sciences, Kyushu University, Fukuoka, Japan; 6 Department of Advanced Environmental Science and Engineering, Faculty of Engineering Sciences, Kyushu University, Fukuoka, Japan; 7 Marine Biosystems Research Center, Chiba University, Chiba, Japan; 8 Department of Biological Science, Tokyo Metropolitan University, Tokyo, Japan

**Keywords:** AMR, AI, Game theory, Social dilemma, Public health, Public health, Psychology and behaviour, Infectious diseases

## Abstract

Currently, antimicrobial resistance (AMR) poses a major public health challenge. The emergence of AMR, which significantly threatens public health, is primarily due to the overuse of antimicrobial agents. This study explored the possibility that the ethical dilemmas inherent in the context of AMR may hinder the adoption of diagnostic artificial intelligence (AI). We conducted a web survey across eight countries/areas to assess public preference between two hypothetical AI types: one prioritizing individual health and the other considering the global AMR threat. Our results revealed a societal preference for the utilization of both AI types, reflecting a conflict between recognizing the significance of AMR and the desire for individualized treatment. Interestingly, the survey indicated significant gender and age differences in AI preferences, and the majority of respondents opposed the idea of AI standardization in treatment. These findings highlight the challenges of incorporating AI into public health and the necessity of considering public sentiment in addressing global health issues such as AMR.

**Supplementary Information:**

The online version contains supplementary material available at 10.1038/s41598-025-95110-x.

## Introduction

Artificial intelligence (AI) has emerged as a useful tool for solving complex problems in human societies. In particular, in the medical field, AI has led to great progress because it can incorporate a vast amount of electronic patient data^1,2^. AI has already achieved equal or greater diagnostic accuracy than human physicians for some diseases, such as breast cancer lymph node metastases^3^, skin cancer^4^, lung cancer^5^, diabetic retinopathy and diabetic macular edema^6^, and common childhood diseases^7^. There have also been reports that physicians can achieve greater diagnostic accuracy with the assistance of AI^8^. Since antimicrobial resistance (AMR) is a global public health challenge that requires both the appropriate use of antimicrobials and the optimization of infectious disease treatment, diagnostic AI has been suggested as a potential solution^9^. However, in the decision-making process regarding antimicrobial prescriptions, AI has not yet been widely adopted. Howard et al. highlighted ethical concerns regarding the quantifying the effects of antimicrobial treatment, particularly in relation to utilitarian AIs that prioritize the greatest good for the majority over individual needs^9^. In this study, we propose that the social dilemma related to the spread of AMR could hinder the widespread adoption of even highly developed AI diagnostic systems.

One of the major factors contributing to the emergence of AMR is the excessive use of antimicrobials, which poses a significant threat to human health^10–12^. The spread of AMR not only results in an increased number of fatal infections among vulnerable populations but also undermines current medical procedures, such as surgeries and organ transplantations, which rely on the effectiveness of antimicrobials^11–13^. A recent report estimated that in 2019, there were 4.95 million deaths associated with bacterial AMR, including 1.27 million deaths directly linked to bacterial AMR^14^. However, the overuse of antimicrobials, which contributes to the evolution and spread of AMR, remains a significant challenge. Indeed, many studies have reported the overuse of antimicrobials^10,15–20^. However, we cannot blame physicians who use antimicrobials preventively to minimize their patients’ risk of infection^10,15^. A survey of general practitioners (GPs) found that 55% felt pressured—mainly by patients—to prescribe antimicrobials, even when they were uncertain of their necessity^17^. Additionally, 44% admitted to prescribing antimicrobials merely to expedite patient turnover, and 45% acknowledged prescribing them for viral infections, fully aware of their ineffectiveness^17^. Similarly, interviews with primary care physicians revealed that some patients, dissatisfied with not receiving antimicrobials, would seek treatment from private doctors until they obtained a prescription^21^. Physicians also noted that refusing to prescribe antimicrobials often resulted in patients consulting another doctor who would comply with their request^21^. These findings emphasize how patient demand significantly influences the AMR issue, demonstrating that responsibility does not rest solely with physicians who have the authority to prescribe antimicrobials, but also with patients whose expectations and preferences play a crucial role. The fact that some patients actively seek antimicrobial prescriptions beyond medical necessity for their own satisfaction indicates the presence of a social dilemma^22^. While these individuals cannot diagnose themselves, they can still prioritize personal satisfaction in treatment choices. The crucial point is that, whether or not their actions are based on malicious intent, their pursuit of unnecessary antimicrobial prescriptions constitutes free-riding on the cooperative behavior of others who accept not receiving antimicrobials. Therefore, the AMR issue can also be understood as a social dilemma among the general public.

**Fig. 1:**
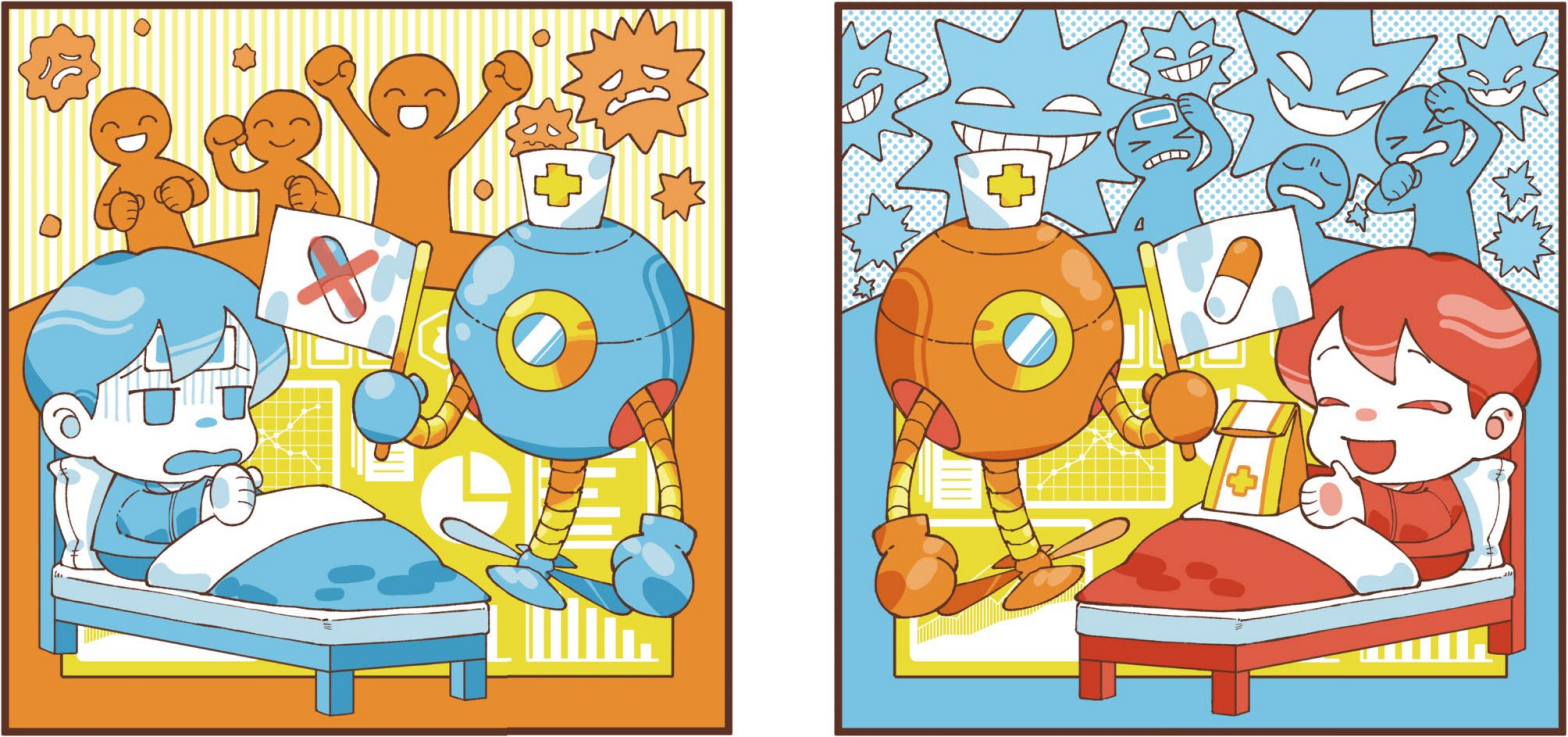
The concept image of World preference AI and Individual preference AI. In the survey scenario, we considered the existence of two types of hypothetical AI systems: ‘World Precedence AI’ (referred to as World-AI) and ‘Individual Precedence AI’ (referred to as Individual-AI). (Left) World-AI is a diagnostic system designed with the aim of reducing the total number of deaths related to AMR from a societal (global) perspective. Therefore, World-AI does not recommend prescribing antimicrobials for rare infectious diseases or for infections that eventually resolve with rest, even if the patient is suffering. (Right) Individual-AI diagnoses and decisions regarding antimicrobial prescriptions purely based on an individual perspective, without considering the global risk of AMR. Consequently, Individual-AI recommends prescribing antimicrobials for preventive purposes and prophylactic prescriptions for rare, accidental infections. Notably, the survey was composed entirely of textual descriptions, and no images of these two types of AI were included in the questionnaire.

Here, we consider the potential of AI-based medical diagnostics in solving the issue of AMR. If AI decision-making is prioritized in diagnostics, the current issue of overuse of antimicrobials might be preventable. However, when introducing diagnostic AI into human society, social dilemmas may become a significant ethical barrier. Social dilemmas arise from conflicts due to discrepancies between the rational preferences of individuals and the optimal choices for society. In the case of AMR, the casual use of antimicrobials tends to be preferred for individuals. In contrast, from a social perspective, such excess antimicrobial use is known to cause AMR. Thus, there may not be a universally convenient type of AI for medical diagnostics to address all concerns. We can consider these two hypothetical diagnostic AI types for different approaches to medical practice: individual versus societal approaches. One type is designed to minimize antimicrobial prescriptions by acknowledging the global threat of AMR (Fig. 1, left), while the other prioritizes individual health and the prescription of antimicrobials without considering the issue of AMR (Fig. 1, right).

We conducted a web survey based on the assumptions of the two hypothetical AI types mentioned above with the following two objectives. First, from the perspective of game theory, we considered the presence of free riders (i.e., selfish and rational individuals who wish to use antimicrobials casually for themselves but want others to refrain from using antimicrobials). The presence of free riders not only supports the existence of a social dilemma surrounding antimicrobial use but also contributes to the factors leading to AMR emergence. The second but more important objective was to investigate public attitudes (public opinion) toward the issue of AMR from a more policy-oriented perspective. In other words, we forced respondents to choose between a society striving to minimize AMR, which might not be optimal for individuals, and a society that prioritizes each individual while ignoring the issue of AMR. This allowed us to test whether a consensus on overcoming the social dilemma and building a society that prevents the spread of AMR could be reached, potentially in the form of a majority decision (e.g., a referendum). In our preliminary study, we conducted an analysis to examine the presence of free riders who prefer to receive diagnosis and treatment from Individual-AI for themselves while expecting others to be diagnosed using World-AI^22^. These free riders accounted for 14.5–27.5% of respondents across the eight surveyed countries/regions, and their presence clearly highlights the existence of a social dilemma^22^. Our ultimate goal is to explore ways to overcome this dilemma by conducting a more detailed analysis of our survey results.

In this study, we focused on the question of which kind of AI should become widespread in society. By exploring whether respondents preferred the adoption of an AI type that is optimal for individuals or a type that is optimal for society, we determined public awareness regarding the introduction of AI diagnostics. Specifically, we asked respondents the following three questions: (1) Adoption preference rates of AIs: Respondents indicated the desired prevalence of World-AI and Individual-AI in society by allocating percentages to each. (2) Agreement or disagreement with standardizing a single diagnostic AI: Respondents expressed whether they supported establishing a unified standard for AI diagnosis, where only one AI type would be adopted, or if they preferred the coexistence of both AI types. (3) Preference in a mandatory standardization scenario: If only one AI type were to remain and the other were to be disabled, respondents selected which AI they would prefer as the sole standard. Through these questions, we analyzed public receptiveness to AI-based medical diagnostics and examined whether individuals prioritize societal benefits or personal interests when considering AI implementation in healthcare.

## Results

The present web survey entitled ‘Survey on Medical Advancement’ collected responses from 41,978 people in 8 countries/areas, including Japan, the United States (US), the United Kingdom (UK), Sweden, Taiwan, Australia, Brazil, and Russia (Table S1). The survey was conducted in four phases: the first phase was conducted in January 2020 in Japan; the second phase was conducted in July 2020 in Japan, the US, and the UK; the third phase was conducted in May 2021 in Sweden, Taiwan, and Australia; and the fourth phase was conducted in June 2021 in Brazil and Russia. We conducted two Japanese surveys before and during the COVID-19 pandemic, expecting differences in public recognition of social dilemmas in public health. In Japan, the US, the UK, Sweden, Brazil, and Russia, a total of 5000 responses (2500 male and 2500 female) were obtained from individuals in their 20–60 s. This included 500 males and 500 females in each age group (20 s, 30 s, 40 s, 50 s, 60 s). In Taiwan and Australia, due to their smaller population sizes, we collected 2,500 responses from individuals with the same age and gender classification.

**Fig. 2:**
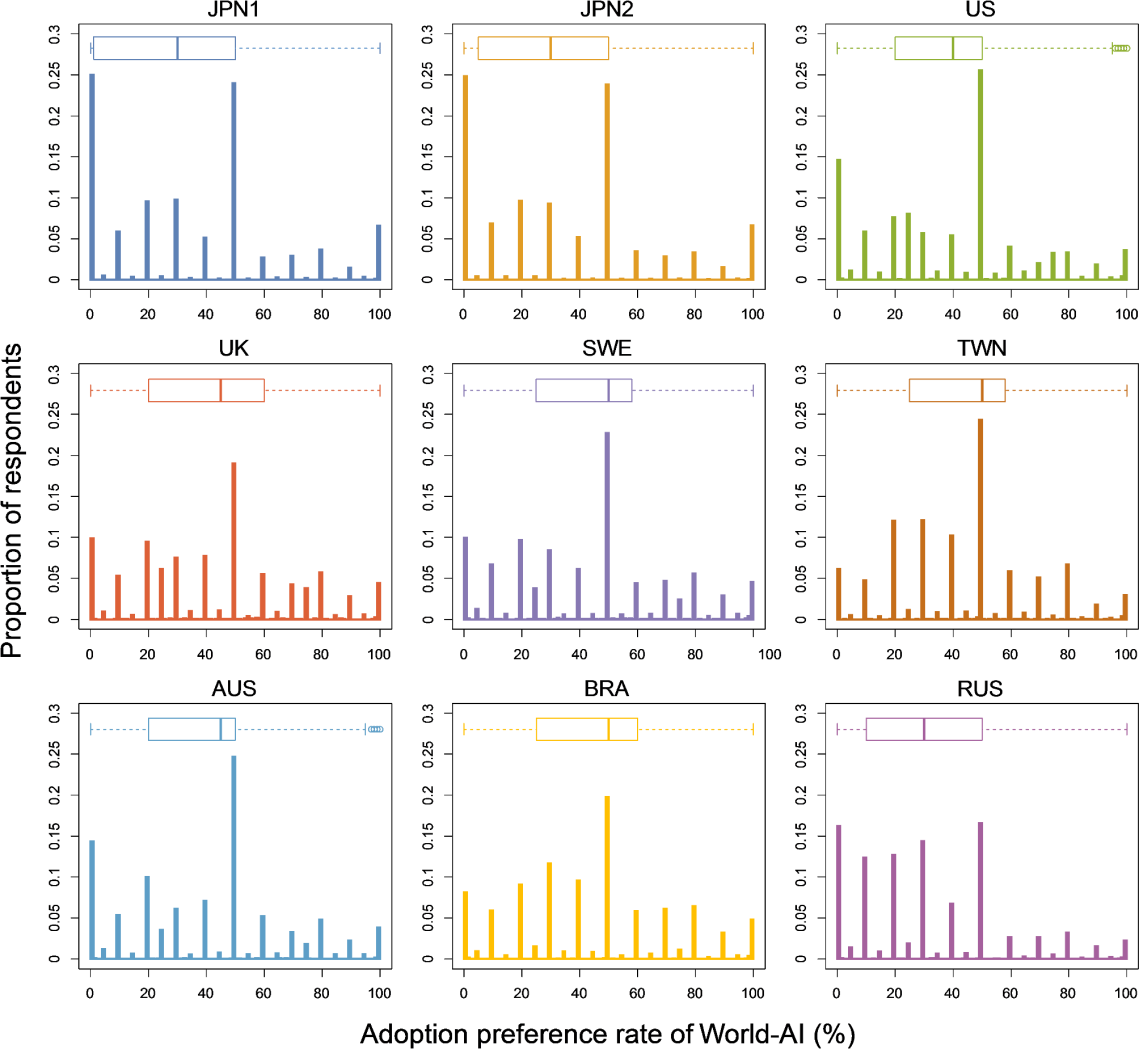
The frequency distribution of the adoption preference rates of World precedence AI in each country/area. When the adoption preference rates of World-AI was X (%), the adoption rate of Individual-AI was 100 – X (%). The responses are concentrated around round numbers such as 0, 10, 20…, 90 and 100 (%). The median is indicated by the box plot at the top of the histogram. If a real vote was to be conducted and the same distribution as this survey was realized, the median voter theorem states that the second quartile (median) would be achieved.

We determined the adoption preference rates of World-AI and Individual-AI (Fig. 2). Note that, when the adoption preference rate of World-AI was X (%); thus the adoption rate of Individual-AI was 100 – X (%). The proportion of respondents from each country/area who answered ‘0%’ (i.e., only Individual-AI is adopted) when asked about their preference level for the adoption of World-AI ranged from 5.8–24.9%, while that of respondents who answered ‘100%’ (i.e., only World-AI is adopted) ranged from 2.3–6.6% (Table S2). In all countries/areas, the majority of respondents expressed a preference for a society that adopts the use of both types of AI (i.e., those who responded with ‘1–99%’), constituting 68.6–91.2% of respondents. Interestingly, in countries/areas other than Japan, ‘50%’ was the most common response, accounting for 16.6–25.6% of respondents. Notably, in Japan, the ‘0%’ response was the most frequent, and the proportions of respondents in the first survey in Japan (JPN1: 24.9%) and the second survey in Japan (JPN2: 24.7%) who selected this response were relatively high compared to those in other countries/areas. Moreover, the proportion of respondents who answered ‘100%’ was also relatively high in Japan, at 6.6%, compared to that in other countries/areas.

**Fig. 3:**
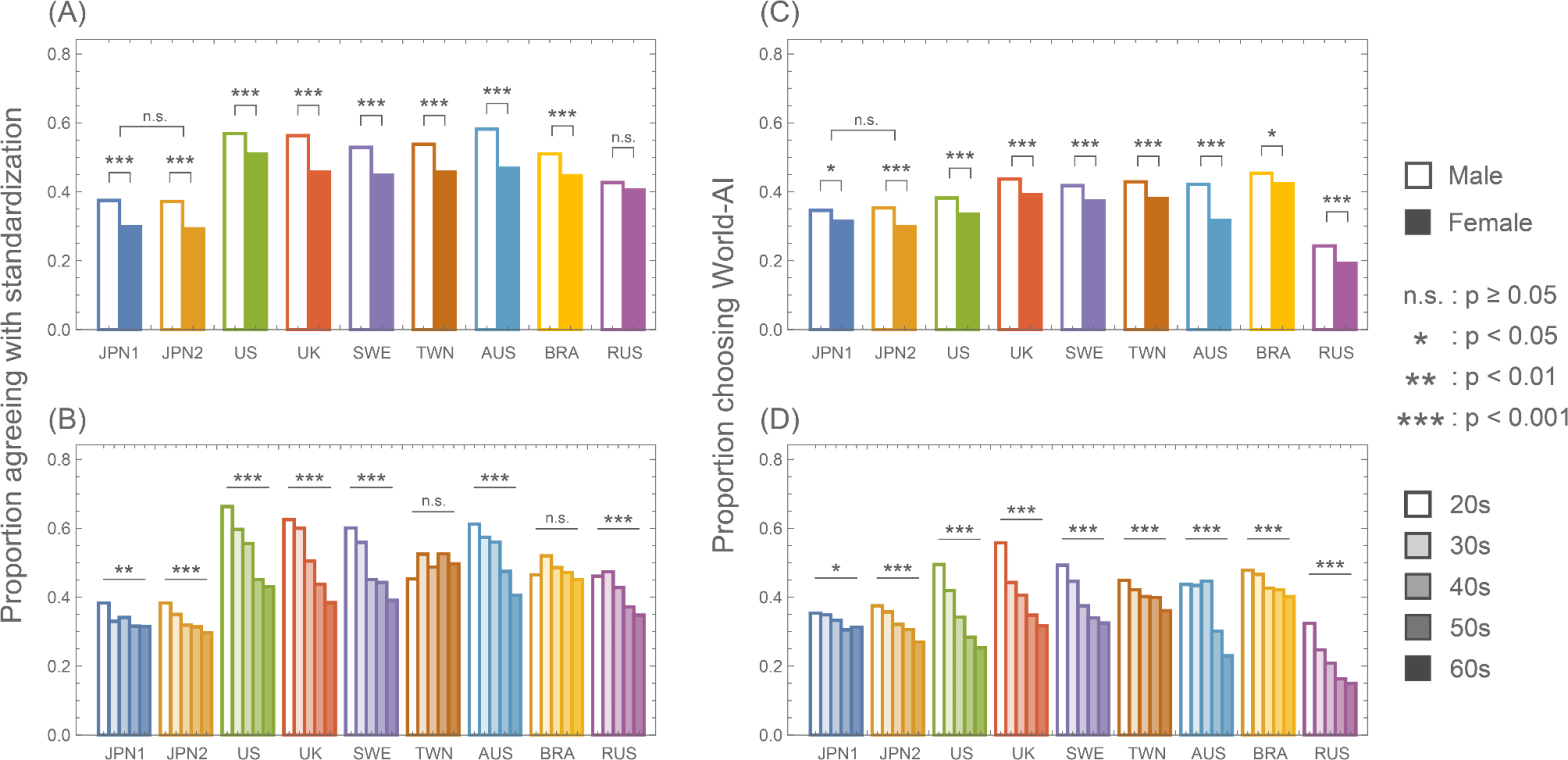
Gender and age group differences in attitudes toward the adoption of diagnostic AI. (**A**, **B**) Proportion of respondents in favor of standardizing diagnostic AI, (**A**) gender group difference and (**B**) age group difference. (**C**, **D**) Proportion of respondents who chose the World precedence AI for standardization, (**C**) gender difference and (**D**) age group difference. The gender differences in attitudes toward standardization and AI preferences were examined using Pearson’s chi-square test with Yates’ continuity correction. Age group differences were analyzed using logistic regression analysis.

Regarding attitudes toward standardization (adopting the use of only one type of AI), 33.3–54.0% of respondents in each country/area held a favorable stance (Fig. 3A,B, Table S3). Note that respondents in favor of standardization include supporters of both World-AI and Individual-AI. The greatest support was observed in the US (54.0%), the UK (51.0%), and Australia (52.5%), while the lowest support was observed in Japan (JPN2: 33.3%). Across all countries/areas, a greater proportion of males (37.2–58.2%) supported for the standardization of one AI type than did females (29.3–51.0%) (Fig. 3A). In countries/areas other than Russia, females were significantly less inclined toward standardization than males were (Figs. 3A and S1). In countries/areas other than Taiwan and Brazil, older respondents tended to be significantly less supportive of standardization than younger respondents (Figs. 3B and S2).

When examining the preference for either of the two AI types, in cases where only one type had to be adopted as the unified standard (with the use of the other AI type being prohibited), the majority of respondents in all countries/areas preferred the Individual-AI (Figs. 3C,D). The proportion of respondents who preferred the World-AI was lowest in Russia (21.8%) and highest in Brazil (43.9%). Notably, females exhibited a significantly lower inclination toward the World-AI than did males (Figs. 3C and S1). Furthermore, older respondents demonstrated a significantly lower preference for World-AI than did younger respondents (Figs. 3D and S2).

The COVID-19 pandemic has had a significant impact on public health awareness. Therefore, it was necessary to examine whether the pandemic influenced respondents’ response trends. Considering its potential impact on the current survey, which also addresses public health issues, we evaluated whether such changes actually occurred. This verification was made possible by utilizing data from Japan, which was collected fortuitously before the outbreak of COVID-19. By comparing the results of the surveys conducted in Japan (i.e., JPN1 and JPN2), we examined the changes in citizens’ perceptions of social dilemmas due to the COVID-19 pandemic. These surveys in Japan revealed no temporal differences in the effects of the COVID-19 pandemic (Figs. 3A,C). These two surveys indicate the stability of public opinion in the present questionnaires.

**Fig. 4:**
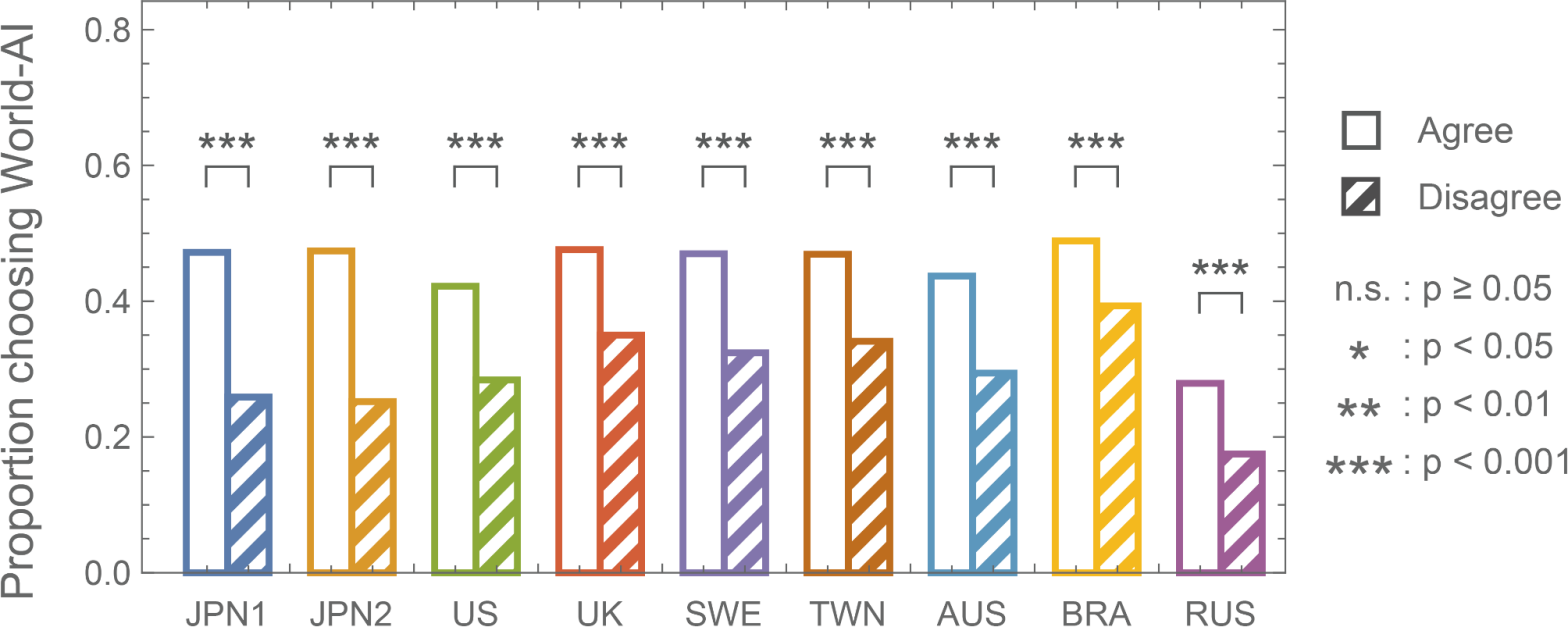
Agreement or disagreement with standardizing a single diagnostic AI system and the proportion of those who chose World precedence AI as the standardized AI system. (White bar) The proportion of respondents who favored the standardization of AI and chose World precedence AI. (Shaded colored bar) The proportion of respondents who were against the standardization of AI and chose World precedence AI. Differences in AI preferences based on attitudes toward standardization were examined by Pearson’s chi-square test with Yates’ continuity correction.

**Fig. 5:**
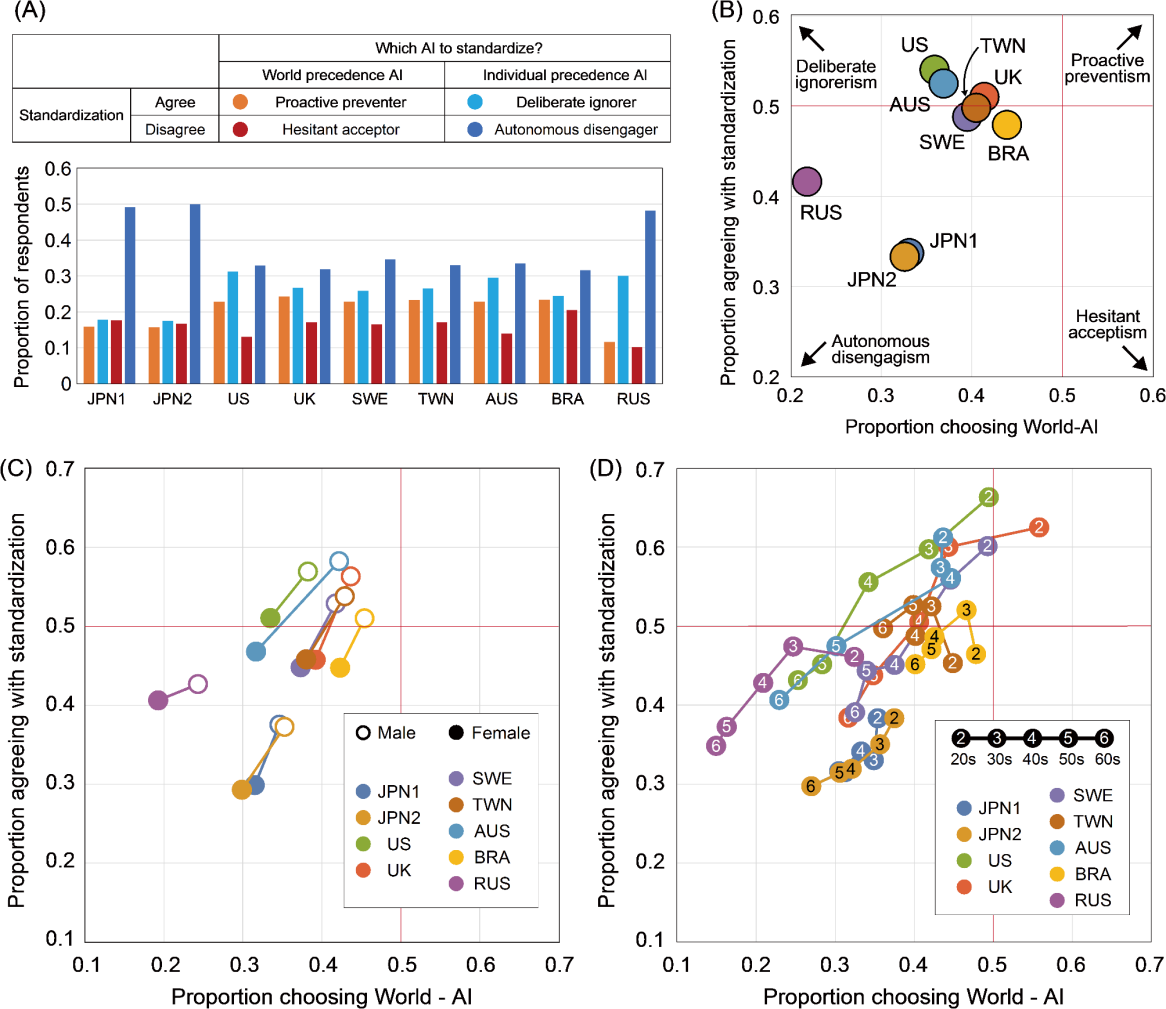
Differences in attitudes toward institutionalization orientation by country/area, gender, and age. (**A**) We divided the respondents into four categories according to their agreement or disagreement with the unified standardization of AI and the type of AI to be standardized. (Dark blue) The most common category of respondents was *autonomous disengagers*, who opposed uniform standardization of a single AI system and preferred Individual precedence AI when standardization is inevitable. (Light blue) The second most common category was *deliberate ignorers*, who chose to make Individual precedence AI the unified standard. (Orange) The respondents who chose to standardize a World Precedence AI were idealists and considered *proactive preventers*. (Red) The *hesitant acceptors*, who opposed uniform standardization but chose World precedence AI if standardization was inevitable, demonstrated a proactive attitude toward the problem of AMR. (**B–D**) Scatter plot of the proportion of respondents who preferred World-AI as the unified diagnostic system on the horizontal axis and the proportion of respondents who preferred AI standardization on the vertical axis. (**B**) Countries/areas differences, (**C**) gender differences in all eight countries/areas and (**D**) age group differences in all eight countries/areas.

Our results also indicate that attitudes toward the standardization of AI are associated with the type of AI preferred. In other words, compared to those who opposed standardization, respondents in favor of standardization significantly preferred World-AI (Fig. 4 and Table S4). Here, to clarify respondents’ expressions of intent regarding AI adoption, we categorized all respondents into four groups based on their attitudes toward AI standardization and the type of AI they ultimately preferred: (i) *proactive preventers*: those who supported standardization and the adoption of World-AI; (ii) *deliberate ignorers*: those who supported standardization and the adoption of Individual-AI; (iii) *hesitant acceptors*: those who opposed standardization and the adoption of World-AI; and (iv) *autonomous disengagers*: those who opposed standardization and the adoption of Individual-AI (Fig. 5). In this context, *proactive preventers* demonstrated the most proactive stance toward the prevention of AMR emergence and spread. *Hesitant acceptors*, while recognizing the significance of AMR issues, also valued the individual freedom of decision-making in their own medication use. *Deliberate ignorers* aimed to protect individual decision-making freedom through institutional means, while *autonomous disengagers* expressed a staunchly individualist attitude. According to the survey results, in all countries/areas, the largest proportion of respondents were *autonomous disengagers* (31.7–49.9%). *Autonomous disengagers* constituted approximately half of the respondents from Japan (JPN1: 49.1% and JPN2: 49.9%) and Russia (48.2%). The next largest group of respondents were *deliberate ignorers* (17.5–31.2%).

The scatter plots (Fig. 5B–D) display attitudes toward the adoption of diagnostic AI across various countries/areas. The horizontal axis represents the proportion of respondents who favored World-AI as a unified diagnostic system, while the vertical axis shows the proportion of respondents who favored standardizing a single AI diagnostic system. An overview suggests the formation of three groups, Japan, Russia, and others (Fig. 5B), with Japan characterized by its reluctance toward standardization and Russia characterized by its aversion to World-AI. Across all regions, males are higher and to the right on the scatter plots than females are (Fig. 5C), indicating a tendency among males to prefer World-AI and view standardization more favorably. Notably, Japanese males were less supportive of standardization than females in other countries/areas, and Russian males show a stronger aversion to World-AI than females in other countries/areas. When examining age differences, younger respondents, specifically those in their 20 s, are higher and to the right on the scatter plots, suggesting that they are more likely to prefer World-AI and are more open to standardization than older respondents are (Fig. 5D). However, even the most pro-standardization respondents in their 20 s in Japan showed levels of support similar to those of respondents in their 60 s in the UK and Sweden. The highest support for World-AI among Russian respondents in their 20 s was on par with the support levels among respondents in their 40 s in Japan and those in their 60 s in Sweden and the UK.

## Discussion

Our survey succeeded in highlighting the barriers involved in incorporating AI-based decision-making into public health. We found that despite recognizing the social dilemma associated with AMR as a public health issue, respondents preferred a society in which both World-AI and Individual-AI are available. This indicates the complex attitudes of citizens who understand the importance of AMR but also value individual medical needs and freedom of choice in treatment. Moreover, our study highlights a division in public opinion regarding the standardization of AI diagnostics, along with significant differences in AI preferences based on gender and age. These findings provide insights into the ethical challenges and societal acceptance considerations necessary for introducing AI in the medical field.

The respondents’ opinions were divided regarding the standardization of a single AI system. Approximately half of the respondents opposed the idea that treatment guidelines should be determined by standardized AI. Furthermore, approximately half of the respondents in favor of standardization preferred Individual-AI to be the standardized AI. It can be inferred that there is a reluctance toward allowing global issues to overly influence individual diagnoses. As previous studies have indicated, current public sentiment toward AI-based medical diagnostics is a mix of resistance and anticipation^23–25^. Considering our results, even if AI diagnostic technology is developed, it would be difficult to make World-AI a uniform standard for diagnosis through democratic methods, such as a national referendum (voting). This result also indicates that the social consensus to refrain from the excessive use of antimicrobials is not yet widespread in the general public. Note that, however, only the use of World-AI as a unified standard could lead to a fundamental solution for the AMR problem in the situation envisioned by our questionnaire. The reason for this is that even if there are only a few types of Individual-AI, the option for patients to receive diagnoses while ignoring AMR becomes available, creating a situation where patients may gravitate toward Individual-AI^22^. Therefore, the coexistence of World-AI and Individual-AI cannot be a fundamental solution for addressing the problem of AMR.

Here, we consider this study from the perspective of game theory. First, agreement or disagreement with standardization indicates whether respondents accept a game in which both themselves and their opponents are compelled to adopt the same strategy. When an individual can only adopt the same strategy as their opponent, he or she cannot free ride on their opponent’s cooperative behaviors. Therefore, it is understandable that those who favored standardization preferred World-AI compared to those who opposed standardization (Fig. 4). In other words, respondents who did not choose a game that allowed for free-riding desired cooperative behavior from others as well. Next, the preference for standardized AI represents the strategy preference when respondents participate in a game where they and their opponent must adopt the same strategy. Under these conditions, the selection of Individual-AI by a majority means that the preferred strategy is not cooperation but defection. This result suggests that the use of antimicrobials is influenced by game structures such as the prisoner’s dilemma game, where defection is always the optimal strategy. Therefore, if we consider the ‘repertoire of effective antimicrobials’ and ‘public health’ itself as commons (common goods/common resources), this survey demonstrated that a game structure that could lead to the tragedy of the commons due to the unregulated consumption of these shared resources (i.e., antimicrobials) exists. Interestingly, Giubilini proposed taxing certain uses of antimicrobials, particularly for treating minor and self-limiting infections in otherwise healthy individuals, as an ethically justified approach to discouraging unnecessary antimicrobial use and mitigating AMR^26^. From a game theory perspective, such a strategy may serve as a deterrent against defection. Applying this concept to our study, it aligns with the idea of increasing World-AI adoption by setting higher consultation fees for Individual-AI than for World-AI, thereby incentivizing choices that help reduce AMR.

Interestingly, our results show significant gender and age differences in attitudes and preferences toward diagnostic AI. Individual-AI was preferred more by females than by males and by older respondents than by younger respondents, with a tendency to avoid standardization. This may reflect differences in attitudes toward AI rather than public health per se. As previous studies have shown, younger respondents and males might have a better understanding of and more experience with AI than older respondents and females^27,28^. This familiarity with AI could foster a sense of trust in diagnostic AI and contribute to a more proactive stance toward AMR.

We need to carefully discuss the impact that the timing of our survey may have had on the results. To explore potential shifts in public awareness, we carried out two surveys in Japan, one before the COVID-19 pandemic and another during it. Interestingly, our findings revealed no significant changes in public perception across these periods due to the impact of the COVID-19 pandemic. This consistency in public views, as seen in both surveys, highlights the stability of public opinion regarding the issues addressed in our questionnaires. This study also underscores the challenge of raising awareness about AMR, emphasizing the need for comprehensive educational initiatives to increase the understanding of this critical public health concern. Considering the impact of the timing of the survey on the results, we may need to focus not on the onset of the COVID-19 pandemic but rather on the period when generative AI, characterized by the introduction of ChatGPT, became widespread. The adoption of generative AI makes examining whether the trustworthiness and image of AI have changed among the public worthwhile.

We also address the limitations related to the representativeness of the sample that arise from the current survey method. When using web survey companies, questionnaires are only distributed to individuals who are pre-registered as potential respondents with the company, which introduces a significant sampling bias. Additionally, these potential respondents intuitively decide whether to answer the questionnaires based on superficial aspects, such as the attractiveness of the title. Consequently, self-selection bias occurs, with individuals who have strong opinions or particular interests in the topic being more likely to participate. These biases are inevitable, even if researchers distribute the questionnaires independently, due to the limited reach available to researchers. Despite acknowledging these limitations, we recognize the utility of web survey companies due to their ability to provide a large pool of potential respondents and effectively filter out inconsistent or apathetic respondents (for details, see the Recruitment section in Materials and Methods).

Our aim is to understand the preferences of the general public, who may not be well-versed in AMR issues, rather than those of medical professionals who are already familiar with the topic. Since society is not primarily composed of healthcare professionals, it is essential to determine which preferences dominate among the general population. However, we have not assessed the variation in respondents’ knowledge. It remains unclear (1) how much knowledge respondents had about AMR before the survey, (2) how much their understanding improved through the preliminary explanation in the questionnaire, and (3) whether they fully comprehended the questionnaire content. This uncertainty leads us to hypothesize that AI preferences may differ depending on the level of AMR awareness. For future studies, combining a comprehension test on AMR with the current survey could provide valuable insights into the importance of accurate knowledge dissemination in addressing the AMR issue.

Finally, we should discuss who should be the main actor in addressing AMR. One potential objection to the significance of our study might be the suggestion that the issue of AMR could be resolved if physicians, who hold the authority to prescribe antimicrobials, manage prescriptions effectively rather than relying on the general public for prescription management. Indeed, many previous studies focusing on the excessive use of antimicrobials have predominantly regarded prescribers (i.e., physicians, hospitals, and policymakers) as the main actors in proposing solutions to AMR^10–21^. Moreover, there are some game theory-based models and discussions that assume a social dilemma among physicians/hospitals due to AMR^13,29–33^. These represent a significant number of previous studies assuming a ‘provider-driven’ society in healthcare, where antimicrobial prescriptions are decided irrespective of patient consent. However, in current societies where informed consent is established, patients themselves can make decisions about their treatment. Indeed, there are reports that pressure and expectations from patients can encourage the prescription of antimicrobials^17,21,34^. In such ‘patient-driven’ societies, all citizens who could be patients should be treated as stakeholders in the AMR issue. Therefore, public opinions and sentiments about medical infrastructure become as crucial as the intentions of prescribers. Our results clearly demonstrated that public health issues, encapsulating social dilemmas such as those related to AMR, may pose a barrier to the societal adoption of even highly accurate diagnostic AI.

## Conclusion

This study examined public attitudes toward the adoption of diagnostic AIs, particularly World-AI, which minimizes antimicrobial prescriptions by acknowledging the global threat of AMR, and Individual-AI, which prioritizes individual needs without considering AMR. The results revealed strong resistance to full standardization. Males and younger respondents were more open to standardization, while the largest group rejected both standardization and World-AI. The findings suggest that ethical dilemmas and public perception are key barriers to the adoption of diagnostic AI in antimicrobial prescription decision-making.

## Materials and methods

### Concept of the survey

We designed a questionnaire to examine barriers to the adoption of medical diagnostic AI in society by considering two hypothetical AI types, one with an individual perspective and the other with a societal perspective. In this scenario, we envisioned ‘Individual-AI’ and ‘World-AI’ (Fig. 1). Both AI systems autonomously diagnose patients and make decisions on whether to medication should be prescribed. The Individual-AI system diagnoses patients based on an individual perspective and suggests the prescription of antimicrobials for preventive purposes, including all prophylactic prescriptions against rare accidental infections (not yet present and unlikely to occur). In its decision-making process, this system does not consider the global risk of AMR. On the other hand, the World-AI system aims to reduce the total number of deaths related to AMR by considering the global mortality risk. Therefore, World-AI does not suggest prescribing antimicrobials for rare infections or infections that can be cured by rest, even if the patient is suffering. The premise of such total mortality was not explicitly stated in the questionnaire, as we aimed to collect naive and intuitive responses from individuals that did not allow immediate logical deductions while answering. We also designed the questionnaire to feature a medical diagnostic AI instead of a human physician in order to eliminate biases arising from differences in healthcare systems across countries and regions, as well as individual perceptions of physicians. This design prevents respondents from shifting all responsibility for antimicrobial use onto physicians, such as by simply thinking, “I take antibiotics if the doctor prescribes them, and I don’t if they don’t.” The questionnaire web pages used in the present survey can be found in the Supplementary Information.

### Questionnaire

The respondents provided their anonymous personal information (gender and age) and answered the following questions:

I. How common, based on percentage, would you like the World precedence AI and the Individual precedence AI, respectively, to become? The total penetration ratio must be 100%.
  ‘World precedence AI: 0-100%’ and ‘Individual precedence AI: 0-100%’
II. Do you agree with either of them becoming the unified standard of AI diagnosis? Or do you prefer that both AI diagnoses are sustained?
  ‘Agree (Only one of them must become the unified standard)’ or ‘Disagree (You would like to sustain both AI diagnosis)’
III. Which AI diagnosis would you like to see become common if only one of them becomes the unified standard and the other becomes disabled?
  ‘Only the World precedence AI is adopted, and the Individual precedence AI is disabled.’ or ‘Only the Individual precedence AI is adopted, and the World precedence AI is disabled.’

All respondents provided informed consent before completing the questionnaire. The respondents were rewarded with electronic points that could be exchanged for cash, gift certificates, frequent flyer miles and electronic money (e-money) for various services. The exact amount of the electronic point reward is unknown due to the company’s privacy policy.

### Recruitment

The ‘Survey on Medical Advancement’ was conducted across 8 countries/areas. This survey was carried out on 4 separate occasions (see Table S1).

For the two surveys conducted in Japan, Cross Marketing, Inc. (https://www.cross-m.co.jp/en/), an internet survey company, created questionnaire webpages in accordance with our study design. Cross Marketing, Inc., also handled the data collection process. As of April 2020, Cross Marketing Inc. maintained an active panel (survey participants who registered in advance) of 4.79 million individuals, defined as survey participants who had been active within the last year. The questionnaire and response section were hosted on a website, allowing respondents to complete the survey and submit their responses. We obtained 500 submissions for each gender and age group by random sampling from all surveys collected during the survey periods.

The surveys conducted in the other 7 countries/areas (i.e., the US, the UK, Sweden, Taiwan, Australia, Brazil, and Russia) were executed by Cint (https://www.cint.com/). Cint is the world’s largest consumer network for digital survey-based research and is headquartered in Sweden. Cint maintained a survey platform comprising more than 100 million consumer monitors in over 80 countries as of May 2020. For surveys conducted in the US, the UK, Sweden, Taiwan, Australia, Brazil, and Russia, Cint Japan (https://jp.cint.com/), the Japanese distributor of Cint, created translated questionnaire webpages following our study design. Cint Japan also managed the data collection process. We obtained at least 500 (the US, the UK, Sweden, Brazil, Russia) or 250 (Taiwan, Australia) submissions for each gender (male and female) and age group (20 s, 30 s, 40 s, 50 s, and 60 s) by random sampling from all surveys collected during the survey periods.

Note that both companies employed a sampling process to exclude inconsistent or apathetic respondents. For instance, individuals with discrepancies in their responses (e.g., those whose registered age did not match their reported age at the time of the survey) were excluded before the data reached the authors. Furthermore, respondents with notably short response times (i.e., less than 1 min) were excluded from the analysis, as their quick responses may have indicated a lack of careful consideration of the survey questions.

### Ethics

This study was approved by the Ethical Committee of the Institute of Tropical Medicine, Nagasaki University (Approval No. 190619217). All methods were performed in accordance with the relevant guidelines and regulations.

### Statistical analysis

We examined differences in the respondents’ AI preferences by performing Pearson’s chi-square test with Yates’ continuity correction (Figs. 3, 4). We also examined differences in gender and age groups by logistic regression analysis (Fig. 3, Supplementary Figs. S1 and S2). The significance codes are as follows: ‘*’ as *p* < 0.05, ‘**’ as *p* < 0.01, ‘***’ as *p* < 0.001, and ‘n.s.’ for nonsignificant. The software used for each analysis was R (ver. 4.0.2) and R studio.

## Electronic supplementary material

Below is the link to the electronic supplementary material.

Supplementary Material 1

## Data Availability

All data related to this study are available at (10.5061/dryad.5mkkwh7f8).
