# Supplementary material for: Barriers to the widespread adoption of diagnostic artificial intelligence for preventing antimicrobial resistance
Source: Sci Rep. 2025 Apr 16;15:13113. doi: 10.1038/s41598-025-95110-x (PMC12003763; doi:10.1038/s41598-025-95110-x)
Supplement: Supplementary file 1 — Supplementary Material 1 [file 41598_2025_95110_MOESM1_ESM.pdf]

Supplementary material for
Barriers to the widespread adoption of diagnostic artificial intelligence for preventing antimicrobial resistance

Hiromu Ito, Takayuki Wada, Genki Ichinose, Jun Tanimoto, Jin Yoshimura, Taro Yamamoto, Satoru Morita

Methods
Information about survey companies
(1) Cross Marketing, Inc. (https://www.cross-m.co.jp/en/)
For the survey in Japan, an internet survey company, Cross Marketing, Inc., created the questionnaire webpages based on our study design, which is described later. The company also conducted the data collection. As of April 2020, Cross Marketing, Inc., has 4.79 million people as an active panel. Here, the definition of an active panel is a respondent who has replied to a survey within the last year.

(2) Cint Japan (https://www.cint.com/)
Cint is the world's largest consumer network for digital survey-based research. The headquarters of the company is in Sweden. Cint Japan is the Japanese distributor of Cint. Cint maintains a survey platform that holds more than 100 million consumer monitors in over 80 countries as of May 2020. For surveys in the US, the UK, Sweden, Taiwan, Australia, Brazil, and Russia, Cint Japan created translated questionnaire webpages based on our study design. The company also conducted the data collection.

Questionnaire web page
Cross Marketing, Inc., created the Japanese version of the questionnaire webpages. Cint Japan created the translated questionnaire webpages based on our study design. By accessing the following URLs, the actual web pages used for the survey can be viewed.

▼USA
Overview
https://d8aspring.post-survey.com/preview/previewPageAll.php?key=Z2zvoUTO
Demo
https://d8aspring.post-survey.com/preview/index.php?key=Z2zvoUTO&lang_c=ja

▼UK
Overview
https://d8aspring.post-survey.com/preview/previewPageAll.php?key=lQhRORK5
Demo
https://d8aspring.post-survey.com/preview/index.php?key=lQhRORK5&lang_c=ja

▼Sweden
Overview:
https://d8aspring.post-survey.com/preview/previewPageAll.php?key=7EagCMPq
Demo:
https://d8aspring.post-survey.com/preview/index.php?key=7EagCMPq&lang_c=ja

▼Taiwan
Overview:
https://d8aspring.post-survey.com/preview/previewPageAll.php?key=1Sl3dSUJ
Demo:

https://d8aspring.post-survey.com/preview/index.php?key=1Sl3dSUJ&lang_c=ja

▼Australia
Overview:
https://d8aspring.post-survey.com/preview/previewPageAll.php?key=hv6drWm6
Demo:
https://d8aspring.post-survey.com/preview/index.php?key=hv6drWm6&lang_c=ja

▼Brazil
Overview:
https://d8aspring.post-survey.com/preview/previewPageAll.php?key=Ln1Iumxc
Demo:
https://d8aspring.post-survey.com/preview/index.php?key=Ln1Iumxc&lang_c=ja

▼Russia
Overview:
https://d8aspring.post-survey.com/preview/previewPageAll.php?key=s8jOCQ5T
Demo:
https://d8aspring.post-survey.com/preview/index.php?key=s8jOCQ5T&lang_c=ja

Questionnaire layouts (e.g., USA ver.)

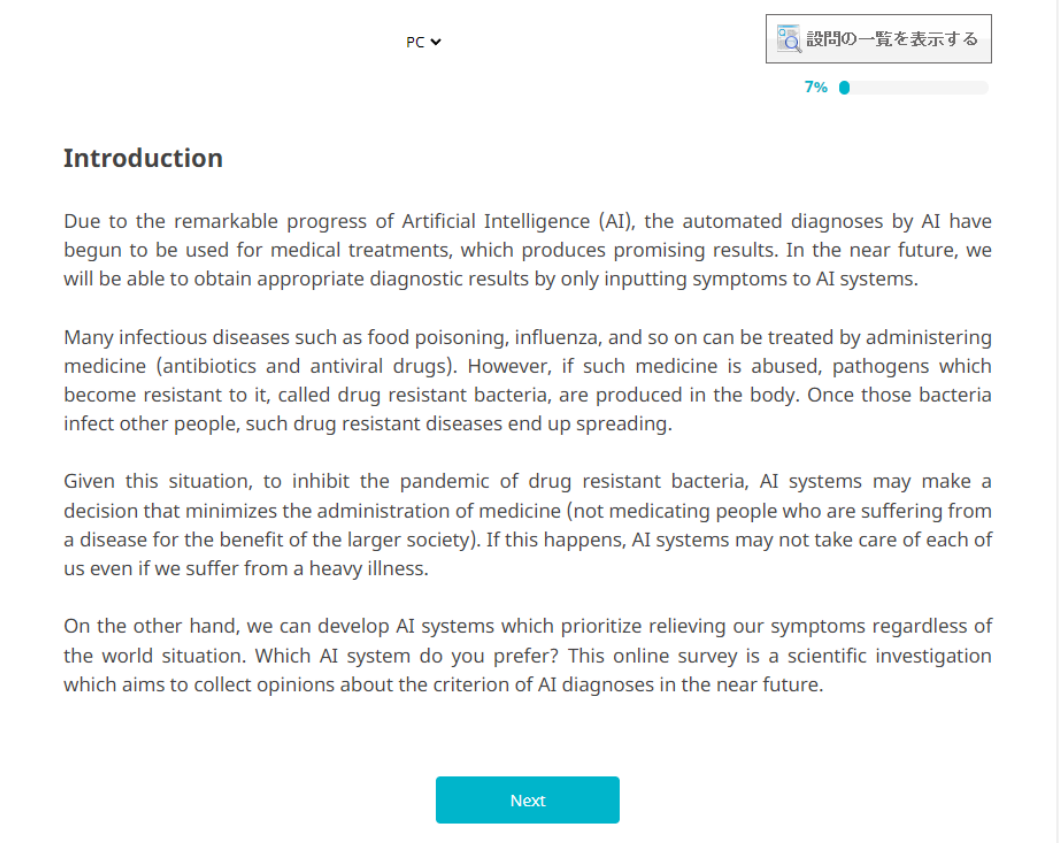

PC

設問の一覧を表示する

7%

Introduction

Due to the remarkable progress of Artificial Intelligence (AI), the automated diagnoses by AI have begun to be used for medical treatments, which produces promising results. In the near future, we will be able to obtain appropriate diagnostic results by only inputting symptoms to AI systems.

Many infectious diseases such as food poisoning, influenza, and so on can be treated by administering medicine (antibiotics and antiviral drugs). However, if such medicine is abused, pathogens which become resistant to it, called drug resistant bacteria, are produced in the body. Once those bacteria infect other people, such drug resistant diseases end up spreading.

Given this situation, to inhibit the pandemic of drug resistant bacteria, AI systems may make a decision that minimizes the administration of medicine (not medicating people who are suffering from a disease for the benefit of the larger society). If this happens, AI systems may not take care of each of us even if we suffer from a heavy illness.

On the other hand, we can develop AI systems which prioritize relieving our symptoms regardless of the world situation. Which AI system do you prefer? This online survey is a scientific investigation which aims to collect opinions about the criterion of AI diagnoses in the near future.

Next

Page 1: Introduction

PC ⌄

設問の一覧を表示する

13%

Confirmation of consent

We would like to know what you think about the criterion of AI diagnoses. Answering the survey takes about 10 minutes. Cooperating with our survey is up to you. When you are answering questions, you can stop answering at any time if you would like to withdraw. Note that you will forfeit your survey incentives for this project in that case.

We do not use the information obtained from the survey other than in this investigation. The web research company destroys the retained data after it delivers the data to us so that personally identifiable information and survey information cannot be recovered. The answer data is stored and analyzed after randomizing the respondent ID not to connect to the respondent. Thus, it is impossible to connect the survey information to personally identifiable information even in the case of an information leak.

This study was approved by the local ethical committee in the Institute of Tropical Medicine Nagasaki University, Japan on July 30, 2019.

I voluntarily agree to participate in this study.

- Yes
- No

Next

Page 2: Confirmation of consent

PC ⌄

設問の一覧を表示する

20%

Scenario

Assume that the AI system has the following two diagnosis criteria:

1) World precedence AI

This AI mainly considers the world situation, but also your symptoms. For instance, drug resistant bacteria prevailing in the world spread by drug abuse. Considering this situation, the AI system may make a decision that it is better for you to sleep at home than prescribing medicine even if you complain of pain. In fact, you would recover from an illness by sleeping at home, but the recovery time may be longer than if you are prescribed medicine. It also raises the risk that your family will be infected.

2) Individual precedence AI

This AI only considers your symptoms and makes the most effective decision for alleviation and treatment of the symptoms. For instance, this AI does not consider the present situation where drug resistant bacteria have spread all over the world. If this AI diagnoses that it is better to prescribe medicine than to sleep at home in order to recover from illness as soon as possible, this AI does so.

Next

Page 3: Explanation of scenario

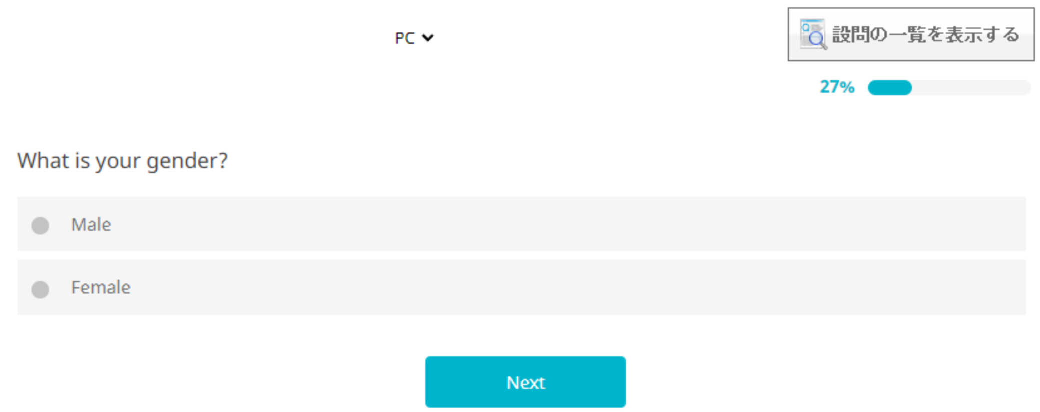

Page 4: Personal information input (gender)

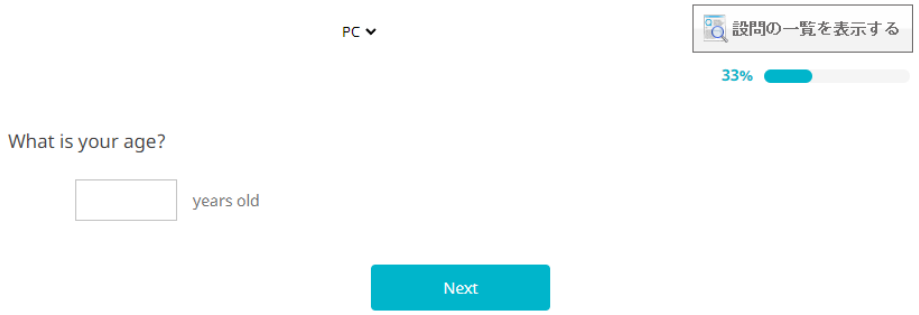

Page 5: Personal information input (age)

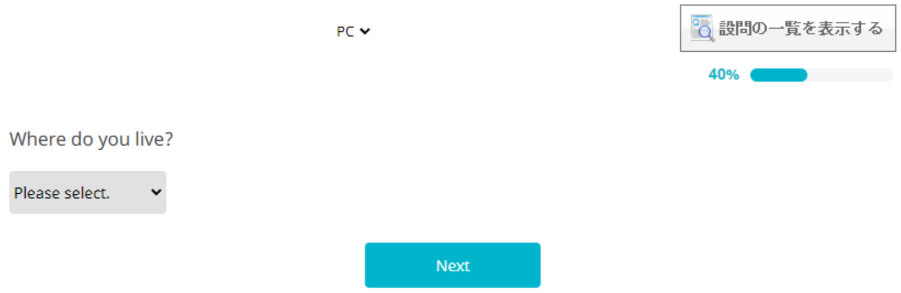

Page 6: Personal information input (state/province level address)

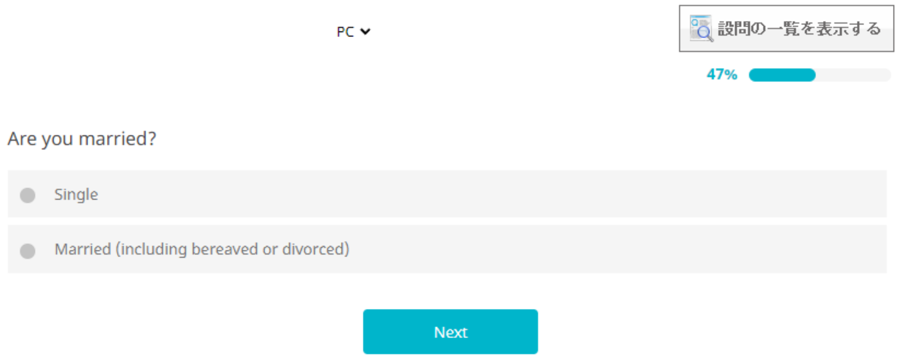

Page 7: Personal information input (marital status)

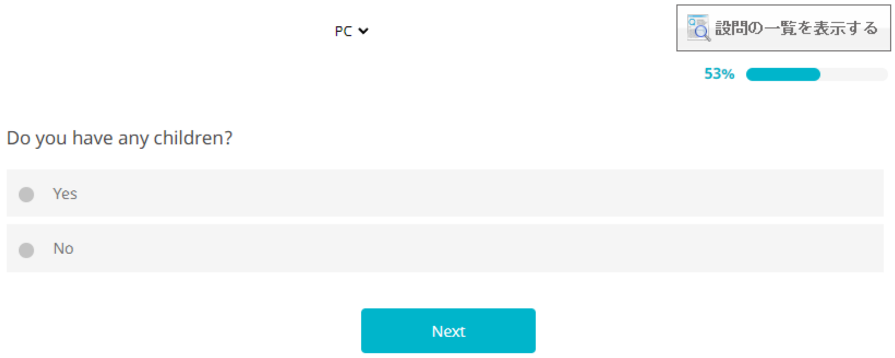

Page 8: Personal information input (existence of children)

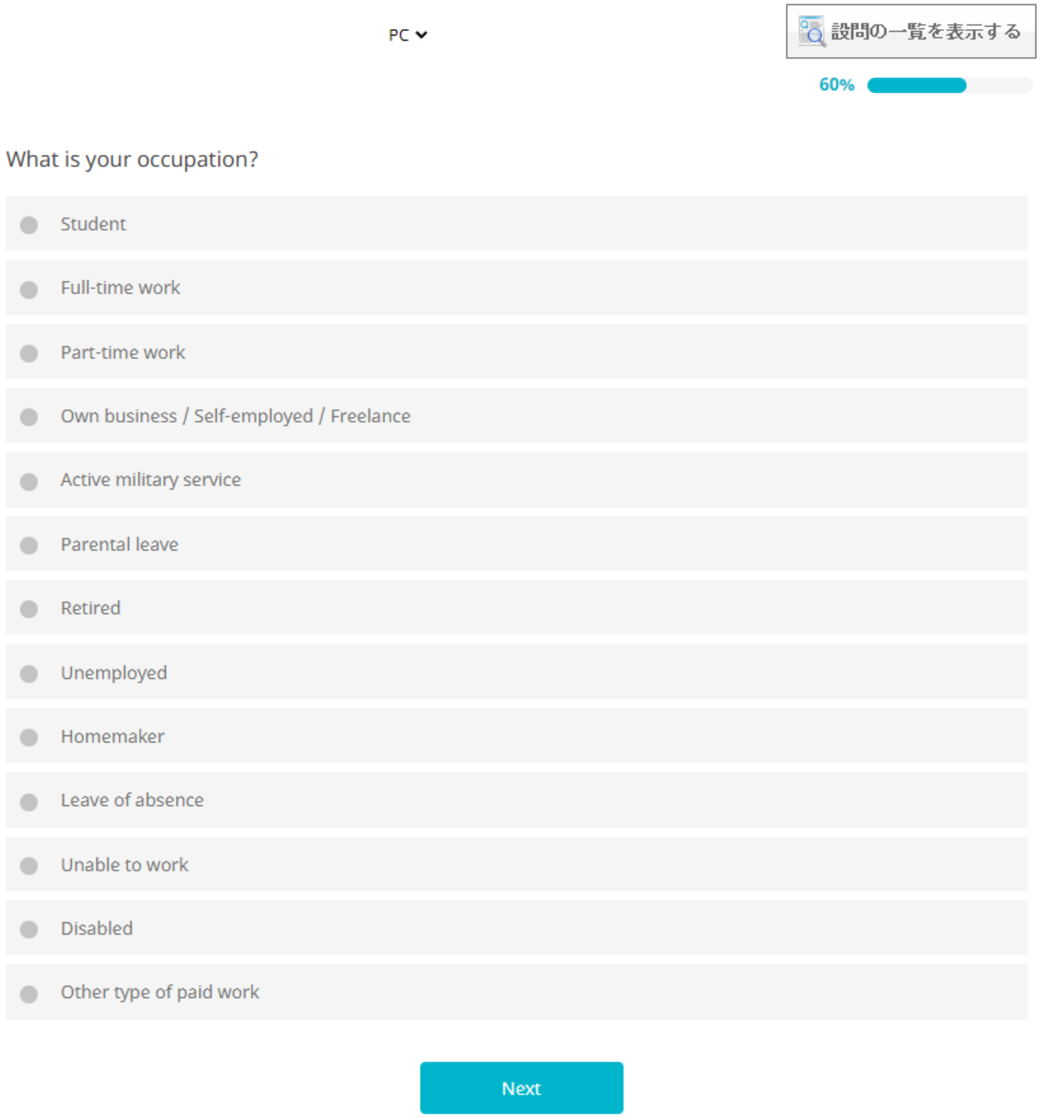

Page 9: Personal information input (occupation type)

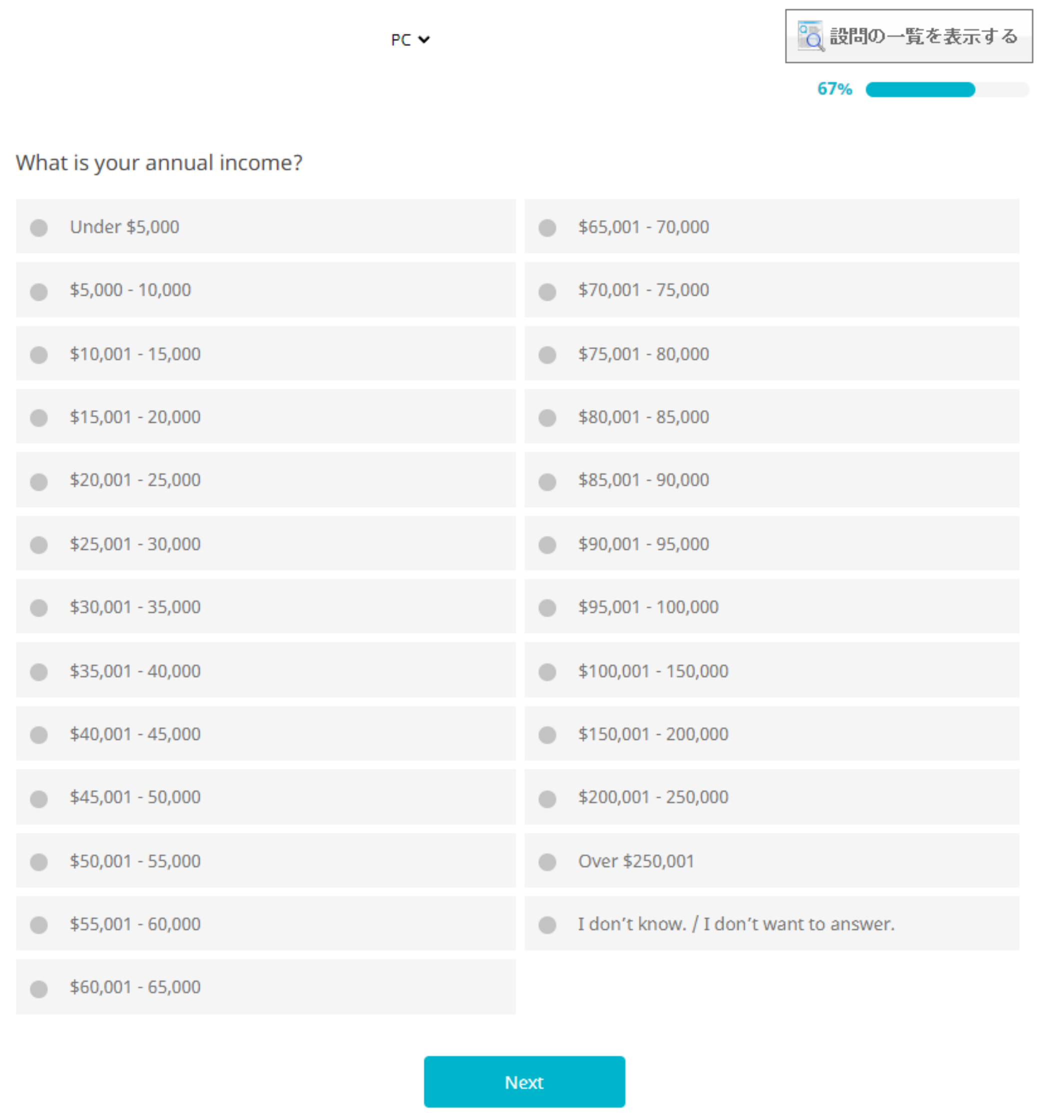

Page 10: Personal information input (income)

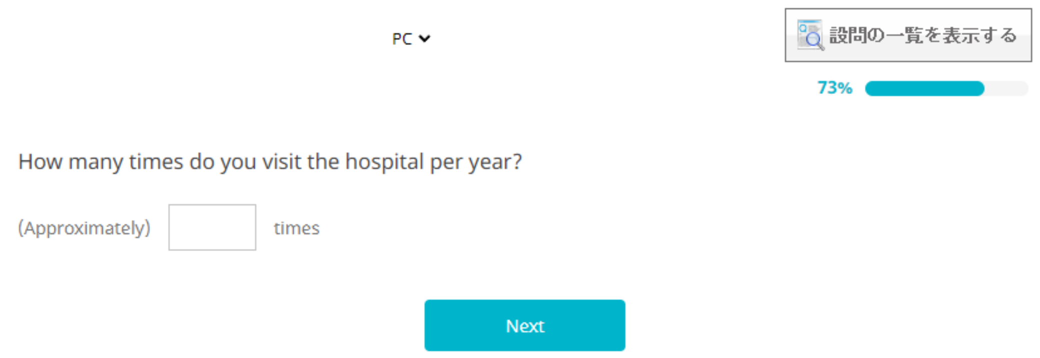

Page 11: Personal information input (frequency of hospital use)

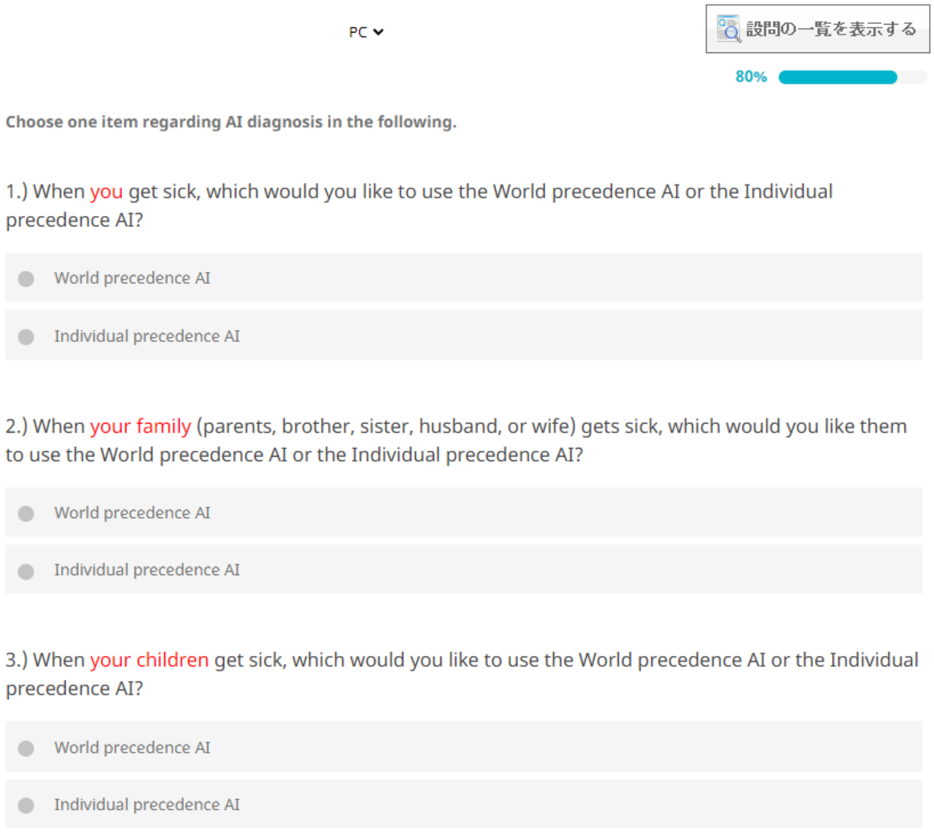

Page 12: AI preference for each diagnostic target

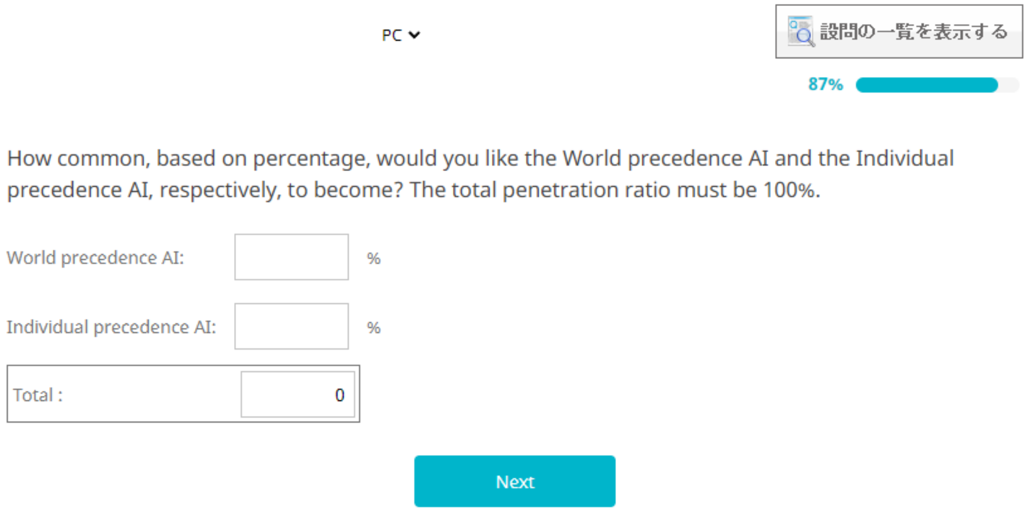

Page 13: AI preference when two types of AI systems are widely used

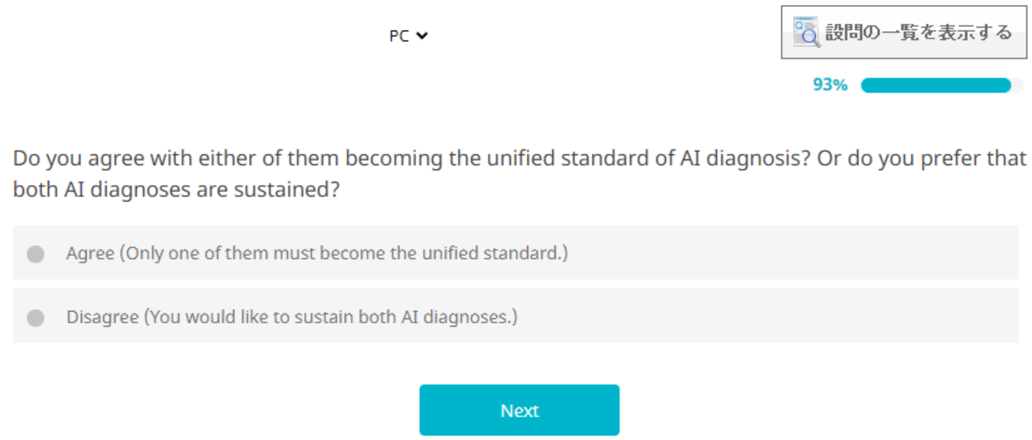

Page 14: Attitudes toward AI standardization

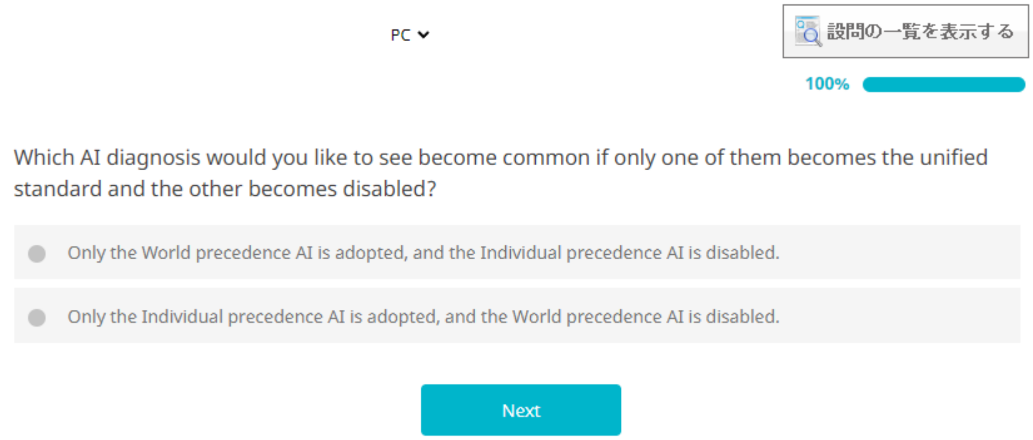

Page 15: Selection of the AI type to be standardized

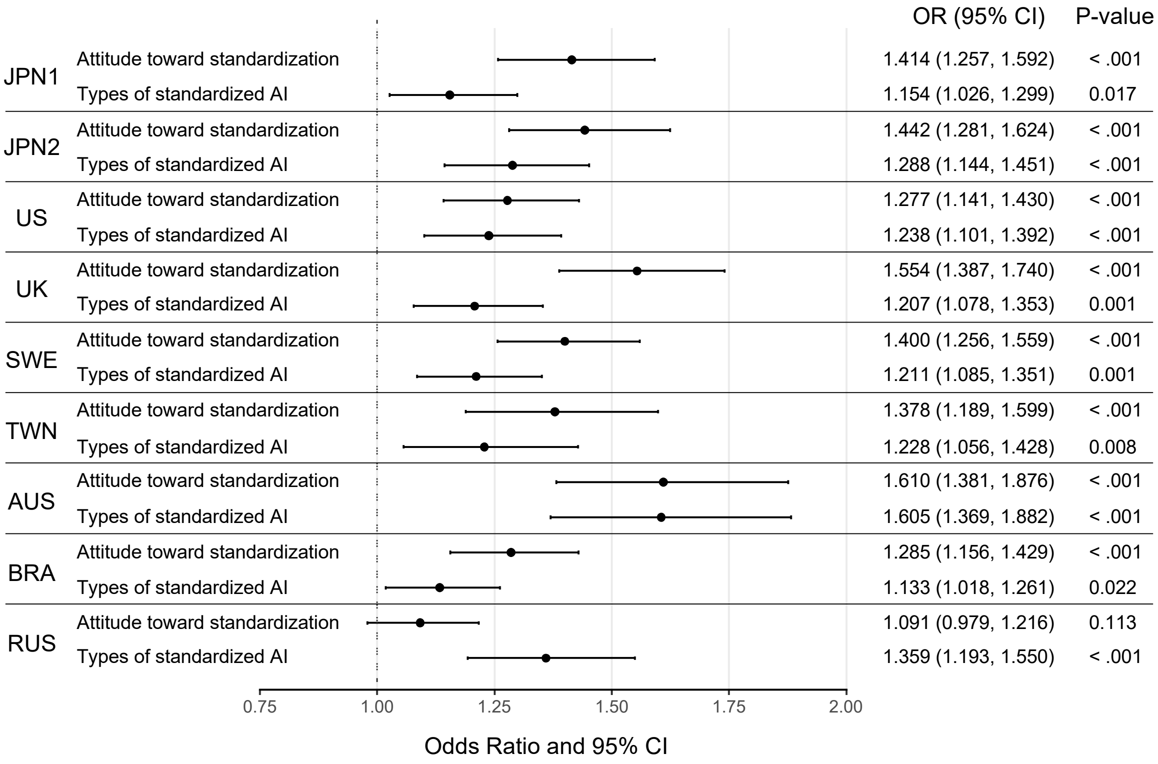

Fig. S1. Odds ratio for logistic regression analysis for the avoidance of AI standardization and preference of Individual-AI by gender.

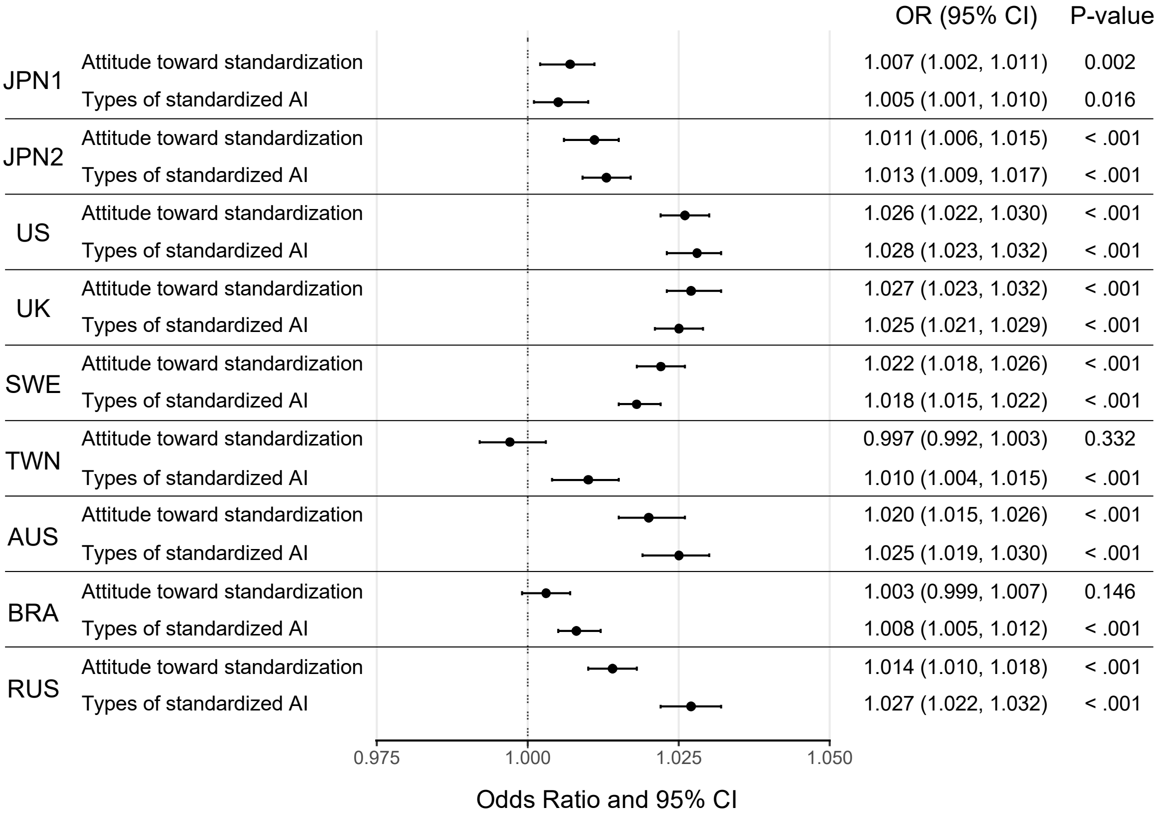

Fig. S2. Odds ratios for logistic regression analysis for the avoidance of AI standardization and preference for Individual-AI by age group.

Table S1.
List of countries and basic information about the survey

Country	Abbr.	Date of Survey	Total sample size (Males:Females)	Survey Company
Japan 1	JPN1	2020/Jan/8~10	5000 (2500: 2500)	Cross Marketing, Inc.
Japan 2	JPN2	2020/Jul/1~7	5000 (2500: 2500)	Cross Marketing, Inc.
USA	US	2020/Jul/1~7	5037 (2519: 2518)	Cint Japan
UK	UK	2020/Jul/1~7	5038 (2517: 2521)	Cint Japan
Sweden	SWE	2021/May/18~26	5446 (2708: 2738)	Cint Japan
Taiwan	TWN	2021/May/18~26	2820 (1405: 1415)	Cint Japan
Australia	AUS	2021/May/18~26	2723 (1353: 1370)	Cint Japan
Brazil	BRA	2021/Jun/23~30	5471 (2726: 2745)	Cint Japan
Russia	RUS	2021/Jun/23~30	5443 (2722: 2721)	Cint Japan

Table S2.
The proportion of the population in each country who preferred World precedence AI (%).

Country	0% (only Individual-AI)	1-99% (both AI types coexist)	100% (only World-AI)
JPN1	1243 (24.9%)	3428 (68.6%)	329 (6.6%)
JPN2	1233 (24.7%)	3435 (68.7%)	332 (6.6%)
US	707 (14.0%)	4148 (82.4%)	182 (3.6%)
UK	479 (9.5%)	4336 (86.1%)	223 (4.4%)
SWE	516 (9.5%)	4682 (86.0%)	248 (4.6%)
TWN	164 (5.8%)	2572 (91.2%)	84 (3.0%)
AUS	378 (13.9%)	2241 (82.3%)	104 (3.8%)
BRA	410 (7.5%)	4799 (87.7%)	262 (4.8%)
RUS	811 (14.9%)	4508 (82.8%)	124 (2.3%)

Table S3.
The proportion of responses related to the standardization of a single AI system in each country (%).

Country	Agree	Disagree
JPN1	1685 (33.7%)	3315 (66.3%)
JPN2	1663 (33.3%)	3337 (66.7%)
US	2719 (54.0%)	2318 (46.0%)
UK	2570 (51.0%)	2468 (49.0%)
SWE	2658 (48.8%)	2788 (51.2%)
TWN	1404 (49.8%)	1416 (50.2%)
AUS	1429 (52.5%)	1294 (47.5%)
BRA	2618 (47.9%)	2853 (52.1%)
RUS	2266 (41.6%)	3177 (58.4%)

Table S4.
The proportion of the population in each of the four institutional orientations (%).

Country	If standardized	Standardization	
		Agree	Disagree
JPN1	World-AI	795 (15.9%)	859 (17.2%)
	Individual-AI	890 (17.8%)	2456 (49.1%)
JPN2	World-AI	789 (15.8%)	840 (16.8%)
	Individual-AI	874 (17.5%)	2497 (49.9%)
US	World-AI	1147 (22.8%)	659 (13.1%)
	Individual-AI	1572 (31.2%)	1659 (32.9%)
UK	World-AI	1223 (24.3%)	865 (17.2%)
	Individual-AI	1347 (26.7%)	1603 (31.8%)
SWE	World-AI	1249 (22.9%)	904 (16.6%)
	Individual-AI	1409 (25.9%)	1884 (34.6%)
TWN	World-AI	658 (23.3%)	483 (17.1%)
	Individual-AI	746 (26.5%)	933 (33.1%)
AUS	World-AI	624 (22.9%)	381 (14.0%)
	Individual-AI	805 (29.6%)	913 (33.5%)
BRA	World-AI	1279 (23.4%)	1121 (20.5%)
	Individual-AI	1339 (24.5%)	1732 (31.7%)
RUS	World-AI	632 (11.6%)	555 (10.2%)
	Individual-AI	1634 (30.0%)	2622 (48.2%)

Supplementary Dataset (separate file).
All data related to this study are available at (doi:10.5061/dryad.5mkkwh7f8)

Data_S1.csv: Responses collected during the first survey in Japan (JPN1)
Data_S2.csv: Responses collected during the second survey in Japan (JPN2)
Data_S3.csv: Responses collected during the survey in the United States (US)
Data_S4.csv: Responses collected during the survey in the United Kingdom (UK)
Data_S5.csv: Responses collected during the survey in Sweden (SWE)
Data_S6.csv: Responses collected during the survey in Taiwan (TWN)
Data_S7.csv: Responses collected during the survey in Australia (AUS)
Data_S8.csv: Responses collected during the survey in Brazil (BRA)
Data_S9.csv: Responses collected during the survey in Russia (RUS).
